# Therapeutic approach to FSGS in children

**DOI:** 10.1007/s00467-006-0310-4

**Published:** 2007-01-01

**Authors:** Debbie S. Gipson, Keisha Gibson, Patrick E. Gipson, Sandra Watkins, Marva Moxey-Mims

**Affiliations:** 1 Chapel Hill School of Medicine, University of North Carolina Kidney Center, University of North Carolina, 7012 Burnett-Womack Hall, CB#7155, Chapel Hill, NC 27599-7155 USA; 2 Children’s Hospital and Regional Medical Center, Seattle, WA USA; 3 National Institutes of Health/National Institute of Diabetes and Digestive and Kidney Diseases, Bethesda, MD USA

**Keywords:** ESRD, Proteinuria, Children, FSGS

## Abstract

Therapy of primary focal segmental glomerulosclerosis (FSGS) in children incorporates conservative management and immunosuppression regimens to control proteinuria and preserve kidney function. In long-term cohort studies in adults and children with primary FSGS, renal survival has been directly associated with degree of proteinuria control. This educational article reviews the current therapeutic approach toward children with primary FSGS.

## Introduction

**Fig. 1:**
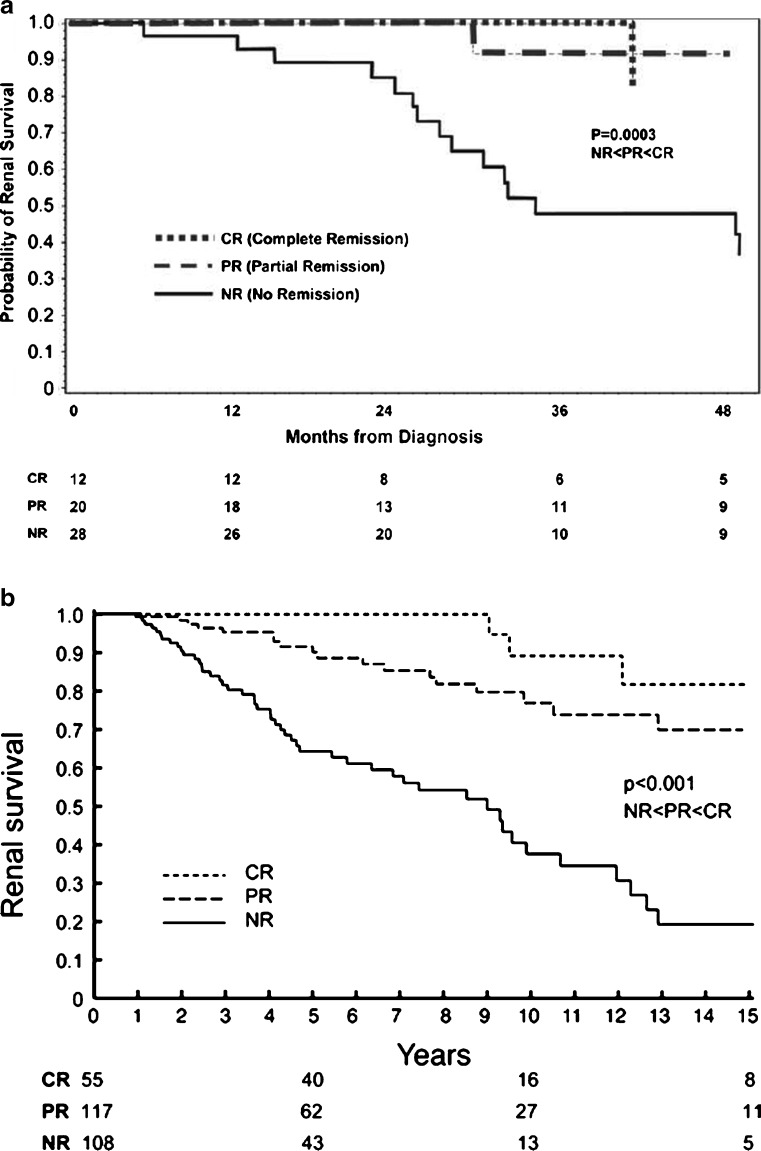
**a** Kaplan–Meyer analysis of the risk of end-stage renal disease (ESRD) by proteinuria remission status in children with primary focal segmental glomerulosclerosis (FSGS). **b** Kidney survival by proteinuria remission status in adults with primary FSGS.* CR* complete remission,* PR* partial remission,* NR* no remission

Focal segmental glomerulosclerosis (FSGS) is a histologic finding that may result from a variety of insults to the kidney. FSGS typically presents with proteinuria and has a high risk of progressive loss of renal function [1]. Treatment of secondary forms of FSGS targets control of the underlying condition. Therapy of primary FSGS incorporates conservative management and immunosuppression regimens to control proteinuria and preserve kidney function. In long-term cohort studies in adults and children with primary FSGS, renal survival has been directly associated with degree of proteinuria control (Fig. 1) [2, 3]. Patients who are resistant to therapies have a significant likelihood of progressing to end-stage renal disease (ESRD) and are a group in need of novel therapies to delay or prevent this outcome [4].

## Genetic considerations

An undefined proportion of patients with classically defined primary FSGS harbor genetic mutations in podocyte-specific genes such as nephrin, podocin, α-actinin-4, and CD2AP [5, 6]. There are conflicting reports about the effectiveness of immune-based therapy in the setting of these mutations, but the likelihood of a positive response may be low [7]. Although mutation-linked cases of FSGS may have a lower response rate to conventional immunomodulatory treatment, these patients still manifest the progressive fibrosis that is observed in nongenetic FSGS.

## Nonimmunosuppressive therapy

### Diuretic therapy

Control of edema in nephrotic syndrome allows not only cosmetic improvement but is expected to decrease pulmonary effusions, decrease ascites, and lower the risk of peritonitis and skin-related problems from edema. Overaggressive diuresis in patients with intravascular depletion may be a risk factor, however, in developing thrombotic complications and acute renal insufficiency.

Loop diuretics are often required for control of edema in patients with proteinuria in the nephrotic range. Delivery of the diuretic to the site of action (lumen of the tubule) is often impaired in nephrotic syndrome due to decreased glomerular filtration rate (GFR), increased binding of the diuretic to intraluminal albumin, and/or decreased delivery of sodium to sites of diuretic activity. An increase of sodium reabsorption in the distal tubule in response to loop diuretic activity may add to resistance to loop diuretics. This distal compensatory mechanism may be diminished by the use of a combination of loop and distal diuretics (thiazides) [8]. Though the addition of aldosterone inhibitors (spironolactone) is theoretically attractive under the theory that edema is in part driven by aldosterone, it is unclear whether spironolactone or other similar medications are clinically helpful to control edema [9–11] An additional advantage to the use of aldosterone inhibitors such as spironolactone is suggested by the antifibrotic properties of these agents, which will be discussed below [12].

Combined albumin and furosemide therapy for anasarca has been studied, as well. Na et al. showed evidence for a mild increase in water diuresis but little evidence that the concomitant use of albumin adds to the natruretic effect of furosemide [13, 14]. Fliser et al. [15] showed a moderate (20%) increase in water and salt excretion when comparing albumin and furosemide to furosemide alone. Haws et al. [16] also showed a mild but transient benefit of albumin and furosemide therapy but commented on the potential serious complications of hypertension, respiratory distress, congestive heart failure, and electrolyte disturbances. Thus, the combination of albumin and furosemide infusions, whether in combination or sequential, may provide a small transient benefit in the therapy of children with severe edema [17].

### Treatment of hyperlipidemia

For patients who become nephrotic from the progression of FSGS, hyperlipidemia is an almost universal finding. Whether the hyperlipidemia associated with nephrotic syndrome should be specifically targeted for treatment in children separately from nephrotic syndrome treatment itself has been a question for more than 20 years. The childhood origin of atherosclerotic disease and increased risk for cardiovascular disease secondary to chronic kidney disease supports an interventional approach.

The report of the expert panel on blood cholesterol levels in children and adolescents [18] from the National Cholesterol Education Program (NCEP) defined categories of hypercholesterolemia in children for total cholesterol and low-density lipoprotein (LDL) cholesterol levels. High levels for total cholesterol were defined as ≥200 mg/dl and for LDL cholesterol as ≥130 mg/dl. Dietary treatment of hyperlipidemia is the first-line intervention. In adults with nephrotic syndrome, soy-based vegetarian diets and supplemented low protein diets have been shown to have potential benefits, decreasing both proteinuria and cholesterol, but have not been shown to slow the decline in GFR [19, 20]. Dietary therapy for dyslipidemia has been effective in reducing lipid levels in children with primary lipid disorders [21].

Based on the report from the NCEP, pharmacologic therapy for children ages 10 years and older should be considered after an adequate trial of diet therapy if LDL cholesterol remains ≥160 mg/dl in children with significant risk for cardiovascular disease, as is seen in children with FSGS. Recommendations for pharmacologic therapy for hyperlipidemia in children from the report suggested that bile acid sequestrants cholestyramine and colestipol should be the first-line agents for treating children with lipid disorders [18]. This was due mainly to concerns about the safety of 3-hydroxy-3-methylglutaryl coenzyme A (HMG CoA) reductase inhibitors (statins) in children. While they are effective in lowering cholesterol and are relatively safe, bile acid sequestrants pose particular problems in children. They are not very palatable, and they may affect absorption of other medications being used, including thiazide diuretics, propranolol, corticosteroids, thyroid hormones, and loop diuretics. A new medication in this category, colesevelam, apparently does not have these problems but is not approved for use in children.

Since the NCEP report in 1992, several studies have been published suggesting that statins are safe in children as young as 4 years of age with familial hypercholesterolemia and do not adversely affect growth, hormone levels, or sexual development [21–23]. Statins are effective in treating the hypercholesterolemia of nephrotic syndrome, with decreases in total cholesterol levels up to 45% [24]. The long-term benefit of statins on renal function may be positive. Down stream from glomerular injury, high levels of urinary protein pass to the renal tubule and are reabsorbed. Protein reabsorption may injure the renal tubule. Statins may inhibit this tubular protein reabsorption and thereby protect from additional renal injury. Whether statins provide renoprotective effects in children has not been well studied, but there are several studies in adults with nondiabetic proteinuria that indicate that statins may slow GFR decline [25]. In these studies, the greatest benefit seemed to accrue in patients with the greatest amount of proteinuria and renal insufficiency. The side effects of statins have mainly been limited to myopathy and hepatotoxicity [26, 27].

Drugs in the fibrate class have also been used alone and in combination to treat hypercholesterolemia in children with nephrotic syndrome [28]. The use of gemfibrozil with a statin may increase the incidence of myopathy. Fenofibrate, approved for adult use in January 2006, may have less interaction due different hepatic metabolism [29]. The long-term safety of fibrates in children has not been well established.

#### Alteration of the renin-angiotensin-aldosterone axis

Blood pressure control for children with FSGS targets values less than or equal to the 90th percentile for age, gender, and height and is consistent with recommendations for all children with kidney disease. Evidence from many trials using angiotensin-converting enzyme inhibitor (ACE-I) and/or angiotensin receptor blocker (ARB) therapy in patients with proteinuria indicate that, beyond their antihypertensive effect, both are effective in reducing proteinuria in a wide variety of renal diseases. Few studies have had significant numbers of patients with FSGS specifically, and fewer still have included children with FSGS. In 1988, Trachtman and Gauthier reported a 50–70% reduction in proteinuria in children with steroid-resistant nephrotic syndrome (SRNS) using ACE-I therapy [30]. Bagga et al. [31], in a randomized, crossover trial of low-dose (0.2 mg/kg) vs. high-dose (0.6 mg/kg) enalapril in 25 patients with SRNS showed dose related responses, with average urine albumin/creatinine ratio reductions of 33% and 52%, respectively. Blood pressure control was similar between the two groups. Several other studies of enalapril and ramipril in children with a variety of proteinuric renal diseases have confirmed the efficacy of these drugs in reducing proteinuria in many, but not all, treated children [32–35]. In the Ramipril Efficacy in Nephropathy (REIN) study, a double-blind study in adults with nondiabetic nephropathy, treatment with ramipril seemed to reduce both proteinuria and the rate of GFR decline more than could be attributed to blood pressure control alone [36]. Wühl et al., in a similar trial in almost 400 children with hypoplasia/dysplasia (70%) and glomerulopathies (13%), had similar findings [37].

There have been many studies in adults with renal disease comparing combined ACE-I/angiotensin receptor blocker (ARB) therapy with monotherapy alone [38, 39]. Though differing in design and findings, overall, the studies seem to indicate greater reduction in proteinuria without a greater frequency of side effects [39–41]. Yang et al. [41] reported greater reduction in proteinuria with combined therapy in a small group of five children with immunoglobulin (Ig)A nephropathy and heavy proteinuria, with no significant side effects noted. The most concerning side effect of ACE-I and ARB therapy is in females of childbearing years, with significant risk of fetal abnormalities reported with in utero exposure [42]. Other side effects of ACE-I therapy were noted in 2.4% of children in one large series with ramipril in children with chronic renal failure [37]. These side effects included decreases in GFR and hemoglobin and increases in serum potassium levels. Acute renal failure, often associated with hypovolemia, has been noted and seems to respond to discontinuation of the medicine until the acute illness resolves. Angioedema and nonproductive cough have been encountered with the use of ACE-I therapy in adults and are less frequently reported in children. ARBs seem to have a lower incidence of angioedema and cough [43, 44]. The incidence of recurrent angioedema in those who experience ACE-I-associated angioedema and are switched to ARB therapy appears to be low [45].

Aldosterone inhibitors have shown potential for alteration of the fibrotic mechanisms in animal models of kidney fibrosis, in the reduction of proteinuria in diabetic nephropathy, and in proteinuric chronic kidney diseases [12, 46, 47]. In the later study of 40 patients with a variety of proteinuric kidney diseases, the combination of ACE-I and aldosterone inhibitor therapy led to a decrease in mean urinary protein excretion in the ten patients assigned to this study arm [48]. Studies in humans with FSGS have not been published.

#### Antioxidants

The potential for antioxidant therapy in FSGS stems from experimental data that supports a role for excessive free radicals in multiple disease states, including chronic kidney disease. Based on antioxidant properties, vitamin E has been evaluated as a potential therapy for FSGS in one small study by Tahzib et al. In this open-label study, 11 children with FSGS were treated with vitamin E for approximately 3 months. A reduction in protein excretion was noted [49]. The antioxidant properties of vitamin E and the relatively low risk for adverse effects of this agent make this an interesting, if unproven, therapeutic option. The combined conservative management approach may control morbidity but leave the majority of patients with uncontrolled urinary protein excretion and risk for progression of disease. The typical approach to FSGS therapy in children is to add immunosuppression at the onset of therapy.

## Immunosuppression

### Corticosteroids

Corticosteroids have long been the mainstay of treatment for childhood nephrotic syndrome, regardless of its etiology. The huge role that these agents play is evident in the way this disease is classified: steroid responsive, steroid dependent, and steroid resistant. The International Study of Kidney Disease in Children (ISKDC) standard dosing has been, and generally continues to be, applied. That is, an 8-week course of oral prednisone at 60 mg/m^2^/day for 4 weeks, followed by 40 mg/m^2^ on alternate days for 4 weeks [50]. Initial ISKDC data showed a corticosteroid response rate of approximately 30% of the 37 children with FSGS studied, and subsequent studies have been consistent in showing a response to oral corticosteroids in a minority of FSGS patients [51]. However, a response to corticosteroids is generally consistent with a more favorable prognosis, even when an initial response is followed by resistance after a subsequent relapse. There are also reported population differences in the response to steroid treatment, such as decreased response in African Americans and Hispanics [52]. Some literature is emerging that shows a lower response rate for patients with the nephrosis 2 homolog, podocin (human) (*NPHS2*) mutation [53]. The more recent debate has been about what steroid, how much steroid, and for how long, before a patient can be definitely declared unresponsive. Certainly, data in adult patients advocate prolonged oral corticosteroid administration, in some cases >6 months [54]. There have been no pediatric trials of prolonged oral corticosteroid use in patients with FSGS. The regimens used vary tremendously, with the most dramatic differences occurring between pediatric nephrologists and internist nephrologists. Due to the generally poor response to the standard oral dosing, some pediatric protocols have advocated high doses of intravenous methylprednisolone, with varying degrees of success [54, 55]. Corticosteroids remain a key component of many therapeutic regimens for FSGS, usually in combination with the various other drugs used to treat this disease, such as alkylating agents or calcineurin inhibitors. Of course, corticosteroid therapy is not without side effects. These include hypertension, growth impairment with prolonged therapy, susceptibility to infection, diabetes mellitus, and osteoporosis [55]. These side effects have led to the tendency toward lower corticosteroid doses and shorter, rather than prolonged, courses.

### Calcineurin inhibitors

#### Cyclosporine A

The rationale behind the initial use of cyclosporine A (CsA) in FSGS and other forms of nephrotic syndrome is the evidence in animal models that the disease may be mediated by lymphokines that mediate glomerular basement membrane damage although it is unclear that this is actually the case. CsA acts on T-helper cells to inhibit interleukin-2 (IL-2) production, cytotoxic T-cell proliferation, and activation of B-cell responses by helper T-cells. However, CsA likely induces remission in proteinuria by two other mechanisms: induction of vasoconstriction of the glomerular afferent arteriole and interference with glomerular basement membrane permselectivity to proteins [56].

It has been two decades since CsA was first reported to show some benefit for patients with idiopathic nephrotic syndrome, especially for patients with steroid-responsive disease who had frequent relapses. One randomized trial of 49 steroid-resistant patients assigned to either CsA or placebo for 6 months showed a benefit in the CsA arm, with a response rate of 70% (complete or partial remission). This is the only medication with documented efficacy for steroid-resistant FSGS in controlled clinical trials in both adults and children [57, 58]. A major concern of long-term CsA treatment is the well-documented potential for nephrotoxicity. Another is the high relapse rate after drug withdrawal. In the Cattran study [57], 60% of the patients who responded to treatment had relapsed by week 78. There is also now concern being raised about secondary resistance developing in patients treated with CsA: an initial induction of remission, relapse when the drug is withdrawn, and resistance on reinstitution of the drug [59]. In this study of 32 children, the diagnosis of FSGS and the presence of C4 or C1q during immunofluorescence staining of kidney tissue appear to correlate with an increased risk of secondary resistance. There are still no guidelines for standardized dosing or duration of treatment of children with steroid-resistant nephrotic syndrome, which probably accounts for the variability in reported response to treatment in the literature.

Side effects of CsA treatment include hypertension, hirsutism, and gingival hyperplasia. As a result of these and the risk for nephrotoxicity, treatment with cyclosporine has not been without controversy in terms of perceived optimal daily dosing, blood level to be maintained, and duration of treatment. A recent Egyptian study in 117 children with nephrotic syndrome, which included 79 patients with FSGS, used low-dose, long-term CsA (more than 2 years of treatment) [60]. The starting dose of 4–5 mg/kg per day was adjusted to maintain a whole-blood trough level of 100–150 ng/ml during the first 2 months and 50–100 ng/ml thereafter. In these subjects with steroid-resistant FSGS, the investigators were able to achieve an almost 70% complete remission rate during the 6 months of therapy. Unfortunately, the relapse rate was substantial upon withdrawal of CsA. Overall, it appears that, as with corticosteroids, a positive response to CsA, even if followed by a relapse, is a good prognostic indicator with regard to the risk for progression to ESRD [57].

#### Tacrolimus

This newer and more potent calcineurin inhibitor has not undergone a controlled clinical trial for the treatment of FSGS, but there are anecdotal reports of responses in patients with nephrotic syndrome, some of whom had FSGS [61]. One retrospective study of 16 children with treatment-resistant nephrotic syndrome, including 13 with FSGS, documented reduction in urinary protein excretion in 13 while on therapy and subsequent relapse in three of the 13 [62]. There are also two small prospective studies in adults that showed a positive response [63, 64]. In these small studies, tacrolimus appeared to present a problem similar to that of CsA, with a majority of patients relapsing on drug withdrawal [63].

### Alkylating agents

DNA alkylating agents such as cyclophosphamide and chlorambucil have been in use since the 1980s for several glomerular diseases, including FSGS. The use of these agents has been limited due to potential side effects, including bone marrow suppression, infertility, hemorrhagic cystitis, and possible future malignancy risk. A retrospective cohort of 29 patients suggested that cyclophosphamide may have some survival benefit in those with at least a partial response measured by impact on proteinuria and progression of chronic kidney disease [65]. A randomized trial in 1996 from the ISKDC evaluated 60 children with FSGS and their response to daily oral cyclophosphamide and alternate day prednisone vs. alternate day prednisone alone. There was no difference in renal survival or proteinuria between the two groups [66]. Due to unfavorable toxic side effects and variable reported efficacy in the literature, alkylating agents are falling out of favor for primary therapy in FSGS.

#### Mycophenolate mofetil

Mycophenolate mofetil (MMF) was introduced in the mid-1990s as an immunosuppressive agent for organ transplantation. Due to its steroid-sparing effect, efforts have been made to expand its clinical application to several glomerular diseases, including FSGS. MMF blocks de novo synthesis of T- and B-cell lymphocytes through noncompetitive, reversible inhibition of inosine monophosphate dehydrogenase. Data on the use of MMF in FSGS has been limited to a few uncontrolled trials with small numbers of patients, but it shows early promise. Choi et al. reported 46 patients with primary glomerulopathies, including 18 patients with FSGS. They found a statistically significant decrease in proteinuria in patients receiving MMF as adjunctive therapy [67]. Cattran et al. reported an open-label, 6-month trial of MMF in 18 patients with steroid-resistant FSGS, 12 of whom were also resistant to alkylating agents and/or calcineurin inhibitors. Although patients did not achieve complete remission, four of 18 had a reduction in proteinuria during therapy [68]. A similar decrease in proteinuria was documented in a series of nine children and young adults with steroid-resistant FSGS who were treated with pulse steroids and MMF [69]. Overall, MMF is showing early promise as a steroid-sparing therapy in FSGS, but questions remain about length of therapy, escalation of dosing, and long-term malignancy risks.

#### Sirolimus

The utility of sirolimus in the treatment of FSGS has been entertained in patients with intolerance or resistance to corticosteroid therapy. One prospective nonrandomized study documented a reduction of proteinuria in 12 of 21 patients treated with 6 months of sirolimus [70]. Conversely, a study of six FSGS patients treated with sirolimus documented a decline in kidney function in five patients. None had a complete remission [71]. In transplant recipients, the use of sirolimus in conjunction with calcineurin inhibitors has also been associated with acute renal failure [72]. Consequently, sirolimus is not recommended for the treatment of FSGS due to the associated renal toxicity.

#### Plasmapheresis

In multiple-drug-resistant primary FSGS, the use of plasmapheresis has been considered a rescue option. The generally accepted rationale is for the removal of a circulating factor from the plasma that alters glomerular barrier function [73]. In primary FSGS in the native kidneys, two small studies encompassing 19 patients reported a response rate between 12% and 55% [74, 75]. The best response was seen using a protocol of plasmapheresis, corticosteroids, and cyclophosphamide, making it difficult to attribute the full response to plasmapheresis alone [75]. A single case report of a child with resistant disease demonstrated improved proteinuria and serum creatinine [76]. At present, plasmapheresis is considered a rescue therapy and is an invasive procedure with significant risks of infection, hypocalcemia, and bleeding. Plasmapheresis is considered an option for prevention or treatment of recurrent FSGS in the transplant recipient based on uncontrolled studies [77, 78].

#### Antifibrotic therapy

There are a large number of patients with multiple-drug-resistant FSGS who are at substantial risk of progression to ESRD and for whom there are no proven therapeutic options. The past decade has witnessed striking advances in understanding the cellular and molecular basis of renal fibrosis and its contribution to progressive kidney failure. Several therapeutic targets have been identified in animal models of fibrosis in the kidney, including molecules involved in the recruitment and activation of mononuclear cells (e.g., chemokines, lymphokines, adhesion molecules), recruitment and activation of interstitial myofibroblasts, fibrogenic molecules [e.g., transforming growth factor (TGF)-β, endothelin-1, angiotensin II, tumor necrosis factor (TNF)-α, and platelet-derived growth factor (PDGF)-β], angiogenic factors [e.g., vascular endothelial growth factor (VEGF)], antiapoptotic molecules, inhibitors of matrix synthesis, and molecules that enhance matrix degradation (matrix-degrading proteases, blockers of protease inhibitors) [79–83]. Crossing many of these mechanisms, peroxisome proliferator activator receptor-γ (PPARγ) agonists alter regulation of renal cell differentiation and proliferation [84–87], extracellular matrix production, macrophage accumulation, tissue inflammation, and apoptosis [88]. The most effective treatment to prevent progression of fibrosis and kidney failure in FSGS is likely to entail a combination of drugs that modulate mediators of fibrosis. The progression of kidney fibrosis is interrupted in part by the use of ACE-I and ARB agents in FSGS therapy. Future therapeutic options are likely to emanate from this area of research.

## Conclusions

Current strategies for control of FSGS use a stepwise approach with a goal of normalization of urinary protein excretion and the prevention of kidney failure. Progress in this field remains a priority in order to prevent the trajectory toward renal failure for patients proven to be resistant to treatment and to identify therapeutic regimens with minimal toxicity.

## CME questions

(Answers appear following the reference list)

1. A 14-year-old boy presents with nephrotic syndrome, normal serum creatinine, and normal blood pressure. He is diagnosed with FSGS by kidney biopsy and treated with corticosteroids. What is the most likely response to corticosteroids in this setting?
  - Complete remission with corticosteroid therapy
  - Dependence on corticosteroids
  - Failure to respond to corticosteroids but improves with cyclosporine
  - Failure to control proteinuria and progression to kidney failure
2. Factors that seem to confer an unfavorable prognosis in children with nephrotic syndrome are:
  - Primary resistance to corticosteroids
  - Resistance to cyclosporine
  - Presence of *NPHS2* podocin mutation
  - African American or Hispanic ethnicity
  - All of the above
3. In the management of FSGS, progressive kidney fibrosis may be slowed by:
  - Furosemide
  - Angiotensin receptor blockade
  - Cholestyramine
  - Prednisone
  - Both b and d
4. FSGS patients with *NPHS2* podocin mutation are more likely to respond to corticosteroids than patients without a podocin mutation (T/F).
5. Cyclophosphamide is considered a mainstay in therapy for FSGS to prevent progression to end-stage renal failure (T/F).
